# Valuing All Voices: refining a trauma-informed, intersectional and critical reflexive framework for patient engagement in health research using a qualitative descriptive approach

**DOI:** 10.1186/s40900-020-00217-2

**Published:** 2020-07-19

**Authors:** P. Roche, C. Shimmin, S. Hickes, M. Khan, O. Sherzoi, E. Wicklund, J. G. Lavoie, S. Hardie, K. D. M. Wittmeier, K. M. Sibley

**Affiliations:** 1 George & Fay Yee Centre for Healthcare Innovation (CHI), 3rd Floor - 753 McDermot Avenue, Winnipeg, Manitoba R3E 0T6 Canada; 2 Department of Community Health Sciences, Max Rady College of Medicine, University of Manitoba, Room S113 – 750 Bannatyne Avenue, Winnipeg, Manitoba R3E 0W3 Canada; 3 Canadian Centre on Disability Studies, Unit #10, 226 Osborne Street North, Winnipeg, Manitoba R3C 1V4 Canada; 4 Ongomiizwin Research, Indigenous Institute of Health and Healing, Rady Faculty of Health Sciences, University of Manitoba, 715 – 727 McDermot Avenue, Winnipeg, Manitoba R3E 3P4 Canada; 5 Department of Pediatrics and Child Health, Max Rady College of Medicine, Rady Faculty of Health Sciences, Health Sciences Centre, University of Manitoba, CE-208 Children’s Hospital, 840 Sherbrook Street, Winnipeg, Manitoba R3A 1S1 Canada; 6 Children’s Hospital Research Institute of Manitoba, 656 – 715 McDermot Avenue, Winnipeg, Manitoba R3E 3P4 Canada

**Keywords:** Patient engagement, Patient and public involvement, Qualitative, Health research, Trauma-informed, Intersectional analysis, Critical reflexive practice, Framework, Engagement, Research partnerships

## Abstract

**Background:**

Critical stakeholder-identified gaps in current health research engagement strategies include the exclusion of voices traditionally less heard and a lack of consideration for the role of trauma in lived experience. Previous work has advocated for a trauma-informed, intersectional, and critical reflexive approach to patient and public involvement in health research. The *Valuing All Voices Framework* embodies these theoretical concepts through four key components: trust, self-awareness, empathy, and relationship building. The goal of this framework is to provide the context for research teams to conduct patient engagement through the use of a social justice and health equity lens, to improve safety and inclusivity in health research. The aim of this study was to revise the proposed *Valuing All Voices Framework* with members of groups whose voices are traditionally less heard in health research.

**Methods:**

A qualitative descriptive approach was used to conduct a thematic analysis of participant input on the proposed framework. Methods were co-developed with a patient co-researcher and community organizations.

**Results:**

Group and individual interviews were held with 18 participants identifying as Inuit; refugee, immigrant, and/or newcomer; and/or as a person with lived experience of a mental health condition. Participants supported the proposed framework and underlying theory. Participant definitions of framework components included characterizations, behaviours, feelings, motivations, and ways to put components into action during engagement. Emphasis was placed on the need for a holistic approach to engagement; focusing on open and honest communication; building trusting relationships that extend beyond the research process; and capacity development for both researchers and patient partners. Participants suggested changes that incorporated some of their definitions; simplified and contextualized proposed component definitions; added a component of “education and communication”; and added a ‘how to’ section for each component. The framework was revised according to participant suggestions and validated through member checking.

**Conclusions:**

The revised *Valuing All Voices Framework* provides guidance for teams looking to employ trauma-informed approaches, intersectional analysis, and critical reflexive practice in the co-development of meaningful, inclusive, and safe engagement strategies.

**Plain English Summary:**

Patient engagement in health research continues to exclude many people who face challenges in accessing healthcare, including (but not limited to) First Nations, Inuit, and Metis people; immigrants, refugees, and newcomers; and people with lived experience of a mental health condition. We proposed a new guide to help researchers engage with patients and members of the public in research decision-making in a meaningful, inclusive, and safe way. We called this the *Valuing All Voices Framework*, and met with people who identify as members of some of these groups to help define the key parts of the framework (trust; self-awareness; empathy; and relationship building), to tell us what they liked and disliked about the proposed framework, and what needed to be changed. Input from participants was used to change the framework, including clarifying definitions of the key parts, adding another key part called “education and communication”, and providing action items so teams can put these key parts into practice.

## Background

Patient engagement (PE) in health research has been defined as the meaningful and active involvement of people with lived experience (referred to throughout as patients, but also includes caregivers, families, friends, and members of the public) in all stages of the research process, including governance, priority-setting, conduct, and knowledge translation [1, 2]. PE strives to create opportunities where all forms of knowledge (including experiential knowledge) are valued equally [3]. Though engagement of patients and members of the public in research is not a novel concept, in the last decade there has been a growing impetus to advance the theory and practice of PE [4], with the aims of improving patient outcomes and care experiences, and decreasing costs to the healthcare system [5]. Demonstrated impacts of PE in health research include improved quality, utility, relevance, acceptability, and appropriateness of research to improve health services and outcomes [6–10]. Other purported impacts of PE include patient empowerment, improved dissemination, uptake of evidence, and fulfilling the moral obligation and fundamental right of patients and other stakeholders to be involved in research that impacts their health and well-being [11]. A 2019 systematic review identified 65 frameworks for supporting PE in health research, categorized as power-focused; priority-setting; study-focused; report-focused; and partnership-focused [12]. These include frameworks developed internationally by government funders and initiatives [1, 13, 14] that include guiding principles (inclusiveness, partnerships, and fairness of opportunity) that emphasize the need to engage with diverse perspectives in health research without discrimination. Many of these frameworks address issues of power imbalance, patient safety, barriers to involvement, and consideration of under-represented groups.

However, through a one-day workshop in 2015 and ongoing discussions with over 50 Manitoba stakeholders (patients, caregivers, community members, community organization leaders, healthcare professionals, researchers, and decision makers), we have heard firsthand about gaps in current PE practice [15]. Specifically, existing PE strategies have been described as continuing to exclude voices traditionally less heard in health research [15]. These are often termed ‘hard-to-reach’ or ‘marginalized’ populations - people living in geographically remote areas, inaccessible environments, or areas with limited transportation, and those who have avoided, disengaged, or been unable to engage with health systems and services due to stigma, negative experiences and/or systemic barriers.^1^ These populations, which include (but are not limited to) Indigenous people, immigrants, refugees, and newcomers, and people with lived experience of mental health conditions, face greater systemic barriers to accessing and receiving healthcare services in Canada [16–19]. Additionally, evidence exists of inequities in patient and public involvement in healthcare improvement strategies both within Canada [20] and abroad [21]. In response to calls to action from Manitoba and beyond, in a 2017 commentary paper Shimmin et al. argued for the need to challenge the status quo and expand existing conceptualizations of PE using a health equity and social justice lens [22]. Shimmin et al. call for the incorporation of trauma-informed practice and intersectional analysis (which includes critical reflexive practice) as a novel and enhanced approach to PE. Born out of the field of social work, trauma-informed approaches involve all parts of an organization seeking to understand how trauma affects the lives of individuals seeking services, including the “vulnerabilities or triggers of trauma survivors that traditional service delivery approaches may exacerbate,” such that programs and services can avoid re-traumatization [23, 24]. Intersectionality is a concept rooted in Black feminism and Critical Race Theory [25] that is increasingly recognized as an important research paradigm for developing a better understanding of the complexity of health inequities [26]. Intersectional analysis can be used to examine (at the micro, meso, and macro structural level) how individuals are shaped by interactions of socially-constructed categories within the larger context of connected structures of power and oppression (e.g. governments, media, public institutions, policies, and legislation) [27]. A central goal of intersectionality is the inclusion of voices traditionally less heard, a critical consideration for the advancement of meaningful PE [28]. Critical reflexive practice refers to the action of critically reflecting on how one’s practical values, assumptions, biases, and actions have been informed by larger systems of power and how they help to uphold, reproduce, and reconstitute oppression and privilege. It includes deconstructing the binary of the personal from the political, and embraces subjectivities as a base for knowledge, employing critical thinking skills that challenge these values, assumptions, biases, and actions [22].

While Shimmin et al.’s work provides an important foundation for the evolution of PE, there is a need to formally incorporate these ideas with existing PE concepts in an organized way that can be used to inform, plan, and carry out PE in practice. A conceptual framework, the end result of bringing together a number of related concepts to explain or predict a given event, or give a broader understanding of the phenomena of interest [29], can help to organize the complex array of issues that need to inform optimal PE. Therefore, the next phase of this work was to develop and refine a framework for PE that builds on established models and re-envisions PE through a health equity and social justice lens by incorporating a trauma-informed intersectional analysis [22].

A draft framework was initially proposed following a 2017 half-day discussion and consensus session with Manitoba community organization leaders, a patient co-researcher, patient engagement and knowledge translation professionals, and academic faculty. This draft framework incorporated the principles of trauma-informed intersectional analysis emphasized by Shimmin et al. with the Canadian Institutes of Health Research Strategy for Patient-Oriented Research (SPOR) *Patient Engagement Framework* [1]. The resulting framework was collectively named the *Valuing All Voices Framework*. An iterative process was used to translate principles in the framework from academic language to more accessible lay language and organize them into categories, or components (Additional file 1). For example, in the context of PE in health research, trauma-informed approaches acknowledge that experiential knowledge may be intertwined with experiences of trauma, and seek to actively resist re-traumatization through the creation of safe spaces. This was translated to “ensuring everyone feels safe and supported.”

The initial *Valuing All Voices Framework* included four proposed components (trust, self-awareness, empathy, and relationship building) (Additional file 1). *Trust* addresses the ‘bearing witness’ aspect of trauma-informed practice and listening to others’ experiences. *Self-awareness* embodies critical reflexive practice and the need for self-care in a trauma-informed approach. *Empathy* addresses how intersectional analysis requires acknowledgement of how systems of power and oppression attempt to divide and ‘other’ people by masking unearned privilege and oppression. Empathy also addresses how trauma-informed practice connects us through the universality of traumatic experiences. *Relationship building* is a critical aspect of PE and knowledge co-production that values lived experience and all forms of knowledge [30]. While the initial framework has a strong theoretical foundation and justification from the existing literature, ensuring that the framework is meaningful and includes the voices of those it is advocating for is essential. The objective of this study was to revise the *Valuing All Voices Framework* through discussions with participants who identify as members of groups whose voices are traditionally less heard in health research (including, but not limited to, First Nations, Inuit, and Metis; immigrants, newcomers, and refugees; and people with lived experience of mental health conditions). Initial groups sought for inclusion in the study were identified based on the Manitoba context, existing relationships with Manitoba community organizations, and practical constraints (limited funds). The specific aims of this study were to (i) determine how participants define the proposed components; (ii) gain insight into how participants view the overall framework in terms of relevance, appropriateness, and usefulness in PE in health research; and (iii) modify the proposed framework based on participant input.

## Methods

### Study design

Prior to recruitment, research team members met with eight local community organizations to identify relevant issues and appropriate interview questions; review proposed methodology, participatory approaches and interview guide (Additional file 2). Community organizations also helped to identify important considerations for potential participants (e.g. supports, meeting locations), and to identify potential recruitment strategies. In response to this input, we employed a qualitative descriptive methodology [31] to collect and analyze data on participant perspectives around the components of the proposed *Valuing All Voices Framework*. A patient co-researcher identifying as an Inuit woman was involved as a research team member throughout the entire study and was involved in planning the study, acquiring funding, data collection, interpretation, and dissemination (oral presentations and manuscript preparation). The nature of the patient co-researcher’s involvement was consistent with the collaboration level described in the International Association for Public Participation (IAP2) spectrum, defined by its goal to “partner with the public in each aspect of the decision including the development of alternatives and the identification of the preferred solution” [32]. The Guidance for Reporting Involvement of Patients and the Public (GRIPP2) short form checklist was used to guide reporting [33].

### Sampling and recruitment

Purposive sampling through community organizations was used to identify potential participants from five key stakeholder groups (First Nations; Inuit; Metis; immigrants, refugees, and newcomers; and people with lived experience of a mental health condition) who face significant barriers to accessing care in Manitoba and whose perspectives are traditionally excluded from health research [34]. Community organizations that serve these groups shared information about the study with their networks and facilitated contact with those interested in participation. Eligible participants included people with lived experience of a health condition (or as a caregiver) who self-identified as a member of one of the key stakeholder groups and were able to provide informed consent. The research team followed recommended processes from the University of Manitoba’s *Framework for Research Engagement with First Nation, Metis, and Inuit Peoples* [35] (Table 1).

**Table 1:** Methods used in recruitment of participants for refining the *Valuing All Voices Framework*

Desired group or individual	Recruitment methods used	Outcomes	References
Patient co-researcher	Partnering recruitment	Inclusion of one patient co-researcher previously engaged by partner organizations	[54]
First Nations	First Nations Research Protocols & Algorithms	Did not proceed past formal application stage (to AMC-HIRGC)	[35]
Metis	Metis Research Protocols & Algorithms	Did not proceed past expression of interest	[35]
Inuit	Inuit Research Protocols & Algorithms	Group interview completed	[35]
Immigrants, refugees & newcomers	Purposive sampling through community organizations (including review of recruitment methods)	Group newcomer interview and five individual immigrant interviews completed	[34]
People with lived experience of a mental health condition	Purposive sampling through community organizations (including review of recruitment methods)	Five individual interviews completed	[34]

### Data collection

**Fig. 1:**
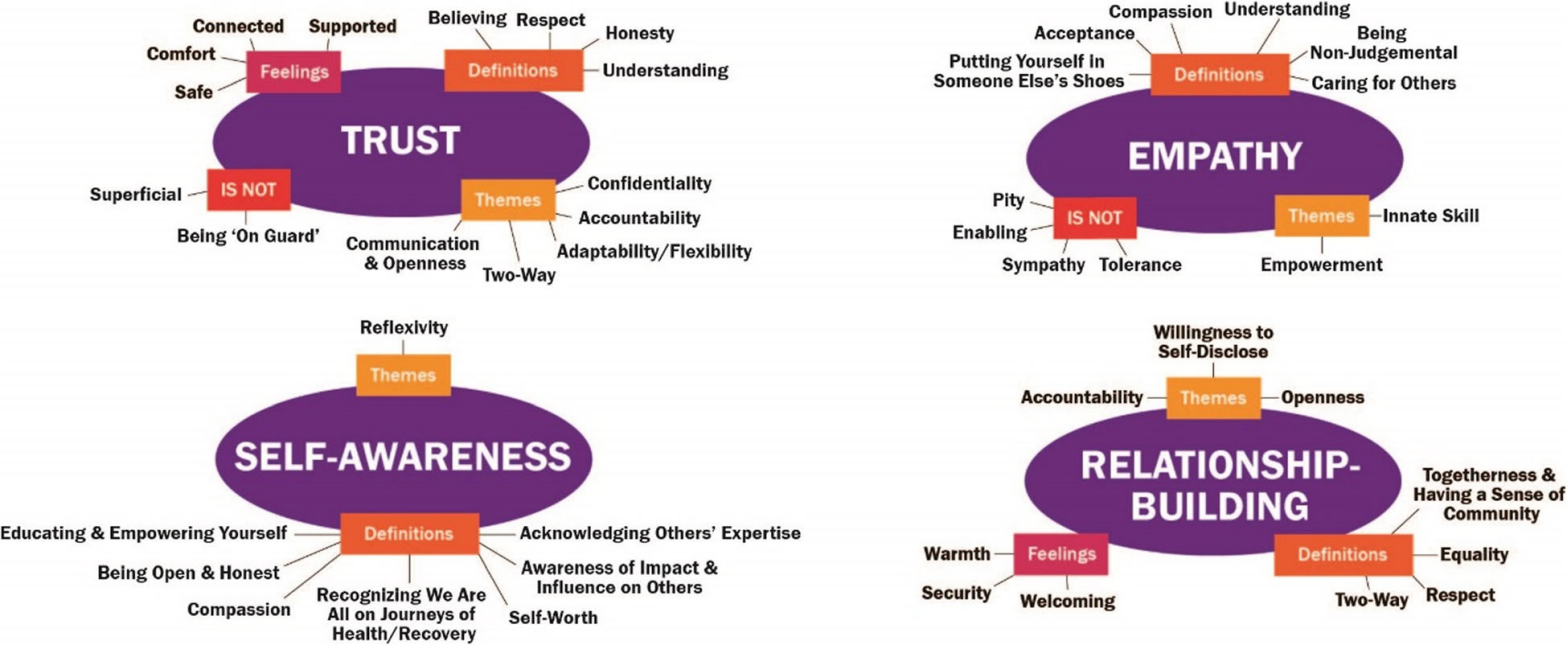
Participant definitions of the four proposed components of the *Valuing All Voices Framework*, represented through word association bubbles

Data were collected through semi-structured group and/or individual interviews, depending on recommendations from community organizations. Group interviews were initially selected as a data collection method as they encourage interaction between participants, and are useful in reflecting the social realities of different groups [36]. The group interview format allows for in-depth exploration and co-construction of participant perspectives, knowledge, and beliefs. However, as recommended by community organizations, to ensure participant comfort, safety, and confidentiality when discussing sensitive issues and experiences, individual interviews were subsequently selected as the method of data collection with immigrant participants and those with lived experience of a mental health condition. Approximately eight participants were invited to group or individual interviews for each group, as is reported to be sufficient to achieve data saturation [37, 38]. Interviews were preceded by introductions. In keeping with the theoretical concepts of trauma-informed and critical reflexive practice, for individual interviews where the interviewer and interviewee had not previously met, introductions were followed by critical reflexive practice questions regarding experiences with healthcare and health research. A discussion of safe spaces was conducted for all interviews. Prior to viewing the proposed framework (Additional file 1), participants provided their own definitions for its four components: trust, self-awareness, empathy, and relationship building (summarized in Fig. 1). Probes were used as needed to clarify and create a deeper understanding of participant experiences and perspectives. Participants were then shown the proposed framework and asked open-ended questions including what they liked and disliked about the proposed framework, and what should be added, removed, or otherwise changed about the framework and its components*.* Framework questions were based on Krueger and Casey’s social analysis approach [39] (Additional file 2). All interviews were audio-recorded and professionally transcribed to ensure accuracy of data. Supports were present as appropriate, including Elders and language interpreters.

Between February and December 2017, two group interviews were conducted: one with three Inuit participants (including a patient co-researcher), two First Nations Elders, and two research team members (CS and TR); and another with six newcomer participants (living in Canada for up to 5 years), three interpreters, and two research team members (CS and OS). Five individual interviews were conducted with people with lived experience of a mental health condition by one or two research team members (CS and TR), identified as MH01-MH05; and four with immigrants and refugees by one team member (OS), identified as IM05-IM08. Input from supports (First Nations Elders and language interpreters) and research team members was excluded from data analysis; however the patient co-researcher acted as both a facilitator and participant for the Inuit group interview. Patient co-researcher contributions included leading discussions with Indigenous community organizations; co-development of methods for involvement of Inuit participants with an Inuit community organization leader; using letters instead of numbers when de-identifying Inuit participants to avoid re-traumatization [40]; convenience sampling in recruitment of Inuit participants; bringing in First Nations Elders who were known to Inuit participants as supports for the Inuit group interview; re-phrasing and addition of questions in the interview guide (Additional file 2); and equal contribution to interpretation of summarized findings and their incorporation in the revised framework.

### Data analysis

A deductive approach to thematic analysis [41] was used to identify codes and themes for participant definitions of framework components and their suggested changes to the proposed framework. Three research team members (PR, MK, OS) read all transcripts, discussed initial thoughts and determined a coding approach. Subsequently, four research team members (PR, MK, KS, KW) open-coded representative transcripts (one from each stakeholder group) and discussed to ensure inter-coder agreement. Two research team members (PR, MK) coded all transcripts, discussed to ensure inter-coder agreement, and devised themes for further analysis. PR conducted a full thematic analysis and kept a reflexive practice journal throughout, to ensure bracketing (attempting to mitigate preconceptions about the work) and maintain audit trails [42]. Retrospective analysis was used to assess data saturation [38], wherein a ‘base’ set of interviews (i.e. the first six) are assessed for how much new information they produce. This number is then used as the denominator in calculating the percentage of new information (unique codes) created in each subsequent interview. By calculating percentages of new information for the remaining interviews (by dividing the number of unique codes produced in that interview by the denominator), the interview that produces a less than 5% threshold of new information can be determined. Any interviews that follow this point of saturation are included as a superscript in reporting. PR or CS contacted participants by email, phone, or in person to review findings and interpretations (member checking) and complete a follow-up demographic questionnaire (Additional file 3). In the member checking stage, participants were provided with a revised version of the framework that incorporated their contributions. Through informal conversation, participants were asked whether they felt their contributions and perspectives were reflected in the refined framework, and provided an opportunity to provide additional changes needed to meet their approval. Quantitative values are reported as mean ± standard deviation.

## Results

### Participants

The study involved 18 individuals who self-identified as belonging to at least one of the following communities/groups: Inuit; immigrants, newcomers, and refugees; and people with lived experience of a mental health condition [15]. Following initial consultations, local and provincial First Nations and Metis organizations declined involvement in the project due to a preference for First Nation-centric research methods, and competing priorities related to funding and self-governance, respectively. Participant recruitment for these groups was not pursued without organizational endorsement. A minimum of eight people from each key stakeholder group were invited to participate; three to six participants from each group completed either a group or individual interview, for a total of 11 interviews. In this study, the first six interviews produced 68 unique codes. By calculating percentages of new information for the remaining five interviews, we found that a less than 5% threshold of new information was reached on the tenth interview (which produced one unique code). Thus, for this study, using the < 5% new information threshold, and a base size 6, data saturation was achieved at 10^+ 1^ interviews.

Of the 18 study participants, we were able to follow-up with nine participants to complete a follow-up demographic questionnaire (Additional file 3), including all five participants with lived experience of a mental health condition; three immigrant or refugee participants; and one Inuit participant. In the member checking stage, only one of the nine participants who completed follow-up requested additional changes, resulting in one new code. The average age of participants who completed the demographic questionnaire was 50.1 ± 15.9 years, ranging from 20 to 75 years. Five identified as women, and four as men. Two participants identified as a person with a disability, and one as deaf/hearing-impaired. One person identified as a member of the LGBTQ2+ community. Two participants identified themselves as immigrants, one as a refugee. Five participants selected ‘other’ for how they identify, and included open-ended descriptions such as “person with a chronic health condition,” “person with lived experience of mental health problems,” “mental depression and cancer,” and “history of depression.” In terms of experiences, six described having a personal health condition requiring some form of support; six had experience caregiving for another person with a health condition; and six had experience accessing healthcare for a health condition (through an emergency room, hospital, walk-in, doctor, or other healthcare professional). Three participants had experienced being unable to access healthcare for a health condition. Four participants reported having previously participated in health research as a participant or patient partner.

### Participant definitions of framework components

Definitions of the proposed *Valuing All Voices Framework* components (trust, self-awareness, empathy, and relationship building) described below are visualized in word association bubbles (Fig. 1). Of all participants, the Inuit and newcomer groups used emotions most often in their definitions. Several participants suggested ‘how to’ action items should be included for the components, and provided rationale for some of the proposed components, though not specifically prompted to do so.

### Trust

Participants defined trust through synonyms (such as “believing”) and related concepts (such as “honesty”). They described the sense of being in a ‘safe space’ – specifically the newcomer group - where people feel comfortable with themselves and their spirituality, and don’t have to worry about re-traumatization. Many described trust as a behavior (as IM07 put it, trust is “a way of life”) and a key component of “two-way” relationships (e.g. accountability and reciprocity). Some interesting insight on trust came from one participant who focused on what trust is *not*– it is not superficial, and if it truly exists, people won’t feel “on guard” (MH05). Participants described *how* trust is built – allowing time, sharing experiences, focusing on resilience and strength (rather than challenges and weakness), and having open communication. One participant emphasized the importance of the explicit use of principles such as the First Nations’ concept of Ownership, Control, Access, and Possession (OCAP™)^2^, even with non-Indigenous people and communities, and providing feedback on research outcomes to participants and partners.

### Self-awareness

The majority of self-awareness definitions spoke to practicing self-reflection^3^ and critical reflexivity, some participants even using the word “reflexivity” verbatim. Participants focused on awareness of both the inner (e.g. values, moods) and outer self (e.g. behaviours, impact on others). Much of the focus of self-awareness was about others – compassion, openness/ honesty, and a sense of responsibility. The newcomer group defined self-awareness as a skill shaped by environmental factors such as culture, religion, incarceration, and health – where one’s ability to be self-aware depends on these factors. In terms of *why* self-awareness is important to PE, one participant mentioned that “a lot of the [time] we don’t think of ourselves as privileged – in comparison to a lot of other people, we are” (IM06). Another participant felt that “self-knowledge increases group or collective knowledge” (IM04), to the benefit of a research team or partnership. Action items to cultivate self-awareness included practicing honesty with one’s self and others, adopting trauma-informed practice, creating safe spaces, and employing cognitive behavioural therapy to address undesirable thought patterns and behaviours.

### Empathy

Participants found empathy the most difficult to define. As one participant stated, “[empathy] is a feeling, not an action – how can you define a feeling?” (MH02). Most described empathy as “understanding” (as an adjective and a verb). Several used the idiom “putting yourself in someone else’s shoes/moccasins” (IM04, IM06, MH01, MH03). Participants who identified as people with lived experience of a mental health condition emphasized the difference between empathy and sympathy/ pity, concepts that are often confused. Several participants described empathy as an innate characteristic – that it must be genuine, and cannot be forced or taught. It also seemed to be linked to morality - that empathy “conjures up the idea that you must care about other people, and maybe that’s not quite it” (MH01). Participants also described what empathy is *not*: it is not enabling, but rather helping people become empowered, and it is acceptance of other cultures, not just tolerance. In terms of *why* empathy is important, one participant stated “it breaks down us vs. them” (MH02) and allows us “to gain greater appreciation for others’ feelings” (MH03). Suggestions for how to cultivate empathy included sharing stories and experiences, and balancing with critical evaluation.

“… as a researcher, that’s a very fine balance – is being empathetic, but also being able to be critically evaluative … the other danger with empathy is that you’re identifying so much with the persons researched – well, for lack of a better word – it’s like getting drawn into their madness.” –MH04.

### Relationship building

Participants defined relationship building in terms of behaviors, feelings, and relationship characteristics (Fig. 1). One participant felt it was too big to explain with “simple words” (IM07). Participants defined successful relationships as being “two-way” (MH04), “equal” (MH02, Inuit group), providing a sense of community, and truly valuing people and their lived experience.

“Sometimes you can do all the research you want, but if you haven’t actually been through what someone else has been through it’s just a different perspective. And I think sometimes as a researcher you have to take a step back and say ‘okay, in this specific instance their perspective is more valuable than mine because [they’ve] gone through it’.” –MH03.

Participants described relationship building as intertwined with the other components, particularly with trust. Relationship building was described by some as a skill set - working with people from different cultures/ backgrounds, assessing and navigating interpersonal situations, and recognizing opportunities for growth and collaboration. One participant described relationship building as a “hierarchy” (IM07), speaking to how relationships between countries, and those in power, influence the potential for relationship building at lower levels. Reasons *why* relationship building is important in PE included the need to feel cared for, creating a “shared vision” (MH04) for research, and improving adherence, compliance, and follow-up with partners and participants. In terms of *how* to build relationships, suggestions included spending time together sharing experiences and leisure activities; open and ongoing communication, honesty, and self-disclosure; and awareness and sensitivity to cultural differences.

### Overall perspectives & changes to the framework

Overall, participants approved of the underlying theoretical concepts of trauma-informed practice, intersectional analysis, and critical reflexive practice, and of the framework itself. The name *Valuing All Voices* was seen as inclusive, though most identified the language used in the proposed definitions (Additional file 1) as inaccessible and too academic. When asked about what to change, participants compared and contrasted their definitions with the proposed framework definitions (Additional file 1). Participants suggested the incorporation of some of their definitions to the components, and in other cases, felt that the proposed definitions were sufficient. Two participants suggested adding a component of education and communication between researchers and patient partners (summarized in Table 2).

**Table 2:** Summary of the refined *Valuing All Voices Framework*. This *Framework*, co-developed with public and patient partners in Manitoba, is intended to provide guidance for research teams seeking to build engagement strategies that are more meaningful, inclusive, and safe. This summary represents stakeholders’ perceptions and definitions of the values important to meaningful and inclusive public and patient engagement and their revisions and feedback on the draft *Valuing All Voices Framework*

Trust	
- Ensuring everyone feels safe and supported	
- Relying on others to care for you	
- Treating people with dignity and respect	
- Believing	
- Loving	
- Cultivating openness and honesty	
- People knowing they can share whatever they need to share	
- Improved when both people have had the same experiences	
- Assuming the best intentions when people appear to be acting difficult or challenging	
- Interpersonal communication and listening	
- Two-way relationships – symbiotic, reciprocal	
- Between family, community, and country; regardless of race or ethnicity	
- Accountability and confidentiality	
How to Practice Trust	
- Allow time to build trust	
- Strengths-based approach (framing challenges positively, focus on resilience)	
- Use principles such as OCAP™ (Ownership, Access, Control, and Possession)	
- Maintain open communication and follow-up with participants and partners	
Self-Awareness	
- Educating yourself	
- Acknowledging privilege and biases	
- Understanding the impact of discrimination based on ethnicity, gender, class, ability, sexuality, age, size, and/or Indigeneity on individuals’ health and well-being	
- Understanding one’s self	
- Being aware of individual physical presence and navigation of surroundings	
- Being aware of one’s own values and internal state	
- Recognizing we are all works in progress, on journeys of health or recovers, understanding where you are on that, and identifying triggers	
- Being aware of power & knowledge imbalances	
- Assessing own liabilities & assets	
How to Practice Self-Awareness	
- Willingness to do work on trauma-informed practice and safety	
- Ensuring support is available (e.g. family)	
Understanding & Acceptance	
- Listening and valuing all perspectives, in order to gain appreciation for others’ feelings	
- Compassion	
- Appreciating resilience: supporting individuals’, families’, communities’, and ethnicities’ ability to overcome challenges of all kinds	
- Acknowledging cultural differences	
- Appreciating the courage and strength of vulnerability (resilience)	
- Genuinely valuing others’ experiences	
- Compassionate understanding without judgement	
- Not to be confused with sympathy or pity	
- Empowerment, not enabling	
- Acceptance, NOT tolerance	
How to Practice Understanding & Acceptance	
- Balance with critical evaluation (to avoid being pulled into negativity)	
- Cognitive behavioural therapy	
- Sharing stories and hearing other’s stories	
- Foster recovery-oriented, strengths-based approaches, which emphasize hope, social inclusion, and community and personal empowerment	
Relationship-Building	
- Acknowledging power imbalances	
- Recognizing opportunities to embrace resistance	
- Understanding the situation	
- Understanding construction of social expectations/structure	
- Understanding different cultural practices	
- Creating a warm & welcoming environment	
- Helping patient & public partners understand the research process, “which mountains can be moved and which ones can’t”	
- Maintaining two-way communication and connection	
- Accountability	
- OCAP (Ownership, Control, Access & Possession)™ principles	
How to Practice Relationship-Building	
- Allow time to develop relationships	
- Spend time together	
- Being open and willing to self-disclose	
Knowledge Sharing, Education & Communication	
- Educating patient and public partners by outlining the process of research; ensuring follow-up and impact; and potential policy and political influence	
- Using different modes of communication for different literacy levels, audiences	
- Engaging early in the process (integrated knowledge translation)	
How to Practice Knowledge Sharing, Education & Communication	
- Outline the research process	
- Discuss expectations	
- Validation (member-checking) of data	

### Trust

Trust was seen as a good, strong word. Suggested changes included adding in something about the universality of trust (at various levels, regardless of social location) and believing that people are generally good –“assuming the best intentions” (MH01). In the context of healthcare and health research, confidentiality was of particular importance to participants, and was added to the revised framework, along with definitions of communication, the two-way nature of trust, and all definitions created in the Inuit group interview (Table 2).

### Self-awareness

In the proposed definition “acknowledging privilege”, participants were divided on the use of the word “privilege”. Some opposed its use, describing it as unclear, and a “big” (IM04) or “horrible” (MH02) word; others appreciated its use. The definition was revised to “acknowledging privilege and biases” for clarity. Other changes included simplification (e.g. changing “understanding the impact of discrimination” to “understanding one’s self”) and specificity around what aspects of the self people should be aware of (both physical and metaphysical).

### Empathy

Suggested changes included valuing and appreciating others – such as “acknowledging cultural differences” (IM05). Many participants suggested clarifying the concept of empathy to ensure its clear distinction from sympathy/ pity. Other suggestions included “balancing empathy with critical evaluation” (MH04), and adding descriptions of what empathy is not - “acceptance, not tolerance” (Inuit group) and “empowerment, not enabling” (MH05). Some participants felt uncomfortable with the title ‘empathy’ itself, suggesting ‘understanding’ and ‘acceptance’ as alternatives; the component title was changed to ‘understanding and acceptance’.

### Relationship building

In the proposed definition “understanding construction of social norms”, the word “norm” was seen as “negative”, or “labeling” (IM05) and participants suggested it be changed to “social expectations” (IM05, MH02). Other suggestions focused on emphasizing communication and accountability.

**Fig. 2:**
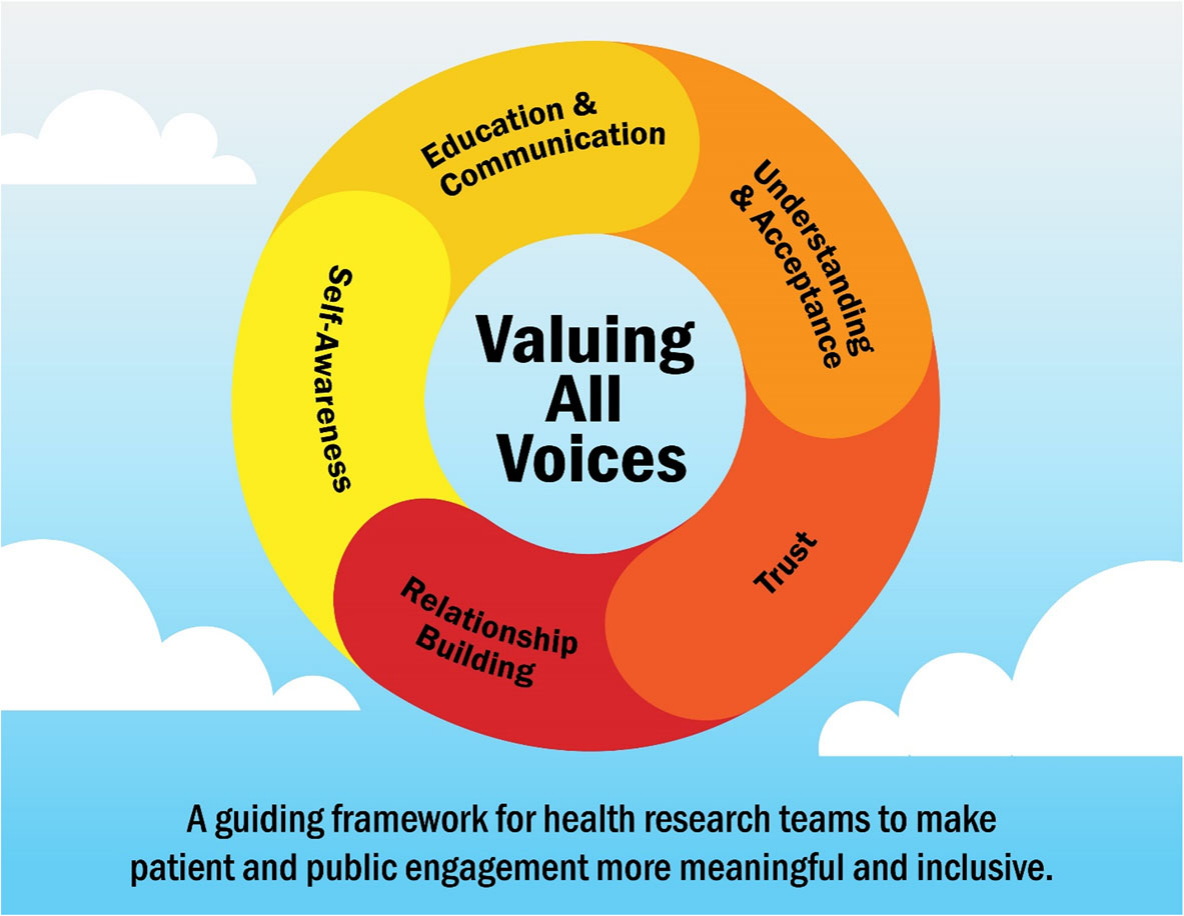
Revised *Valuing All Voices Framework* image with input from study participants

Based on suggestions from participants, participant-created definitions and changes to proposed definitions were incorporated into the revised framework summary (Table 2). Where suggestions from different participants were contrary, the preferences of the majority were used to make revisions. A number of new definitions were added to the framework components, as was a new component of education and knowledge. A few suggestions for minor changes to the visual representation of the framework included increased font size, creating a flow between theoretical concepts and framework components, and graphic illustrations to assist in understanding. These changes are reflected in the revised framework image (Fig. 2) and summary (Table 2).

## Discussion

Participants refined and enriched the *Valuing All Voices Framework* in a number of ways. This included challenging the language used in the proposed framework and clarifying definitions to make them more accessible. Collectively, participant input emphasized the need for a holistic view of engagement that goes beyond the research process, exemplified by the focus on open and honest communication; the creation and maintenance of trusting relationships; and capacity development for both researchers and patient partners to engage fully and meaningfully. In particular, the use of emotions in framework definitions by the Inuit and newcomer groups indicates a focus on the bigger, more important picture of how we relate to one another on a personal level. Participants highlighted the complexity and inter-relatedness of the proposed components, in part through how they seemed to blur the lines in defining and discussing framework components. One example is the focus on clarifying the various “levels” to which definitions for trust and relationship building refer (i.e. personal vs. workplace relationships; trust between individuals, communities, cities, or countries) by members of the newcomer group and participants identifying as immigrants. As the intent of the proposed framework was for use by academic researchers, descriptions of the proposed language as inaccessible for participants was not surprising. This was even more prominent for those whose first language is not English. Although interpreters were present in the group interview with newcomers, concepts such as trust or empathy may not translate directly in their meaning or definition. Thus, time taken to clarify concepts, address power in the research process, and answer critical reflexive practice questions before diving into definitions and suggested changes was an important aspect of the process.

Participant input also identified and refined critical elements of trauma-informed practice and intersectional analysis that are important to people with lived experience, including those whose voices are traditionally less heard in health research. For example, emphasis on aspects of self-worth and compassion for one’s self and others speaks strongly to the principles of trauma-informed practice. Participants added other critical elements related to the foundational theory of the proposed framework, such as using strengths-based approaches and acknowledging resilience; an understanding of the bi-directional nature of relationships, including research partnerships; and the importance of accountability and self-disclosure (i.e. sharing one’s own lived experience) in research relationships. Participants also emphasized the importance of breaking down ‘othering’ in research, and clearly outlining expectations in the early stages of research partnerships. These elements speak to the need for researchers and patient partners to work together to challenge traditional modes of knowledge production and engagement from perspectives that are traditionally less heard in health research.

Of the 65 PE frameworks reviewed by Greenhalgh et al. [12], many represent important contributions to the theory and practice of PE, including addressing power dynamics and providing guidance in evaluation of PE strategies. However, a limited number of studies involve patients or members of the public in their development [45–52], and even fewer report framework development specifically in response to gaps or needs identified by patients and the public themselves [53]. Of those that involve patients and the public, none challenges the status quo by specifically aiming to engage with voices traditionally less heard in health research; nor do they address aspects of trauma or safety in PE. The *Valuing All Voices Framework* complements pre-existing frameworks by delving further into the principles of inclusivity and diversity put forth by participants as actual skills and behaviours required for meaningful engagement of voices traditionally less heard. The *Valuing All Voices Framework* is an important and unique framework for PE in health research in that (i) it was created in response to gaps identified by patients and members of the public; (ii) it has involved patients and members of the public both as members of the research team and as study participants; and (iii) it addresses the need for safety and critical reflexive practice in employing a social justice and health equity lens, through which issues around healthcare, services, and health research can be viewed. The *Valuing All Voices Framework* provides guidance for teams looking to employ trauma-informed approaches, intersectional analysis, and critical reflexive practice in the co-development of meaningful, inclusive, and safe engagement strategies.

### Outcomes and impacts of patient engagement

Positive impacts of engagement included leveraging existing relationships for methods co-development and recruitment; capacity-building for researchers and academics involved regarding communication and decision-making with patient and public partners; as well as inclusion of an Indigenous Inuit non-academic perspective in decisions throughout the research process. Engagement resulted in more relevant and appropriate methods and interpretation of findings, particularly the effective use of storytelling in data collection and sharing of preliminary findings at conferences and keynote lectures. Challenges related to engagement primarily included unanticipated delays, and underestimation of the time and resources required to conduct research-related activities.

### Moving forward

Contributions to the framework around the concept of safety focused primarily on emotional, spiritual, and cultural safety, though we must not neglect other important aspects of safety in PE (psychological and physical). For example, attention to patient and public partners’ physical needs during engagement activities, especially those that require travel or long hours (e.g. availability of foods for people living with diabetes and celiac disease) and accessibility issues (e.g. sign language, support animals, ramps) are aspects often neglected in engagement in research, leading to unsafe situations or exclusion of certain individuals.

### Limitations

Acknowledging challenges inherent in conducting research with voices traditionally less heard in health research, such as extended timeframes, increased costs, and the need for community partnerships [34], feasibility constraints limited data collection to groups identified as relevant to the Manitoba context, and with whom established relationships existed. Despite existing connections, recruitment and retention of participants was hindered by challenges such as geographical distance, resource availability, time constraints, and language barriers. Though the number of participants involved in refining the framework was limited, we did observe saturation within the groups interviewed, as well as for each participant contacted in the member checking stage. However, there are voices missing from the current discussion, which we hope to engage with in the future, including other groups whose voices are traditionally less heard in health research – namely people from the five First Nations language groups represented in Manitoba, Metis people, older adults, people experiencing substance use issues or homelessness, and more members of the LGBTQ2+ community (as only one participant in the study self-identified as a member of the LGBTQ2+ community). Further understanding of the intersections of experience (including intersecting categories of social location and their relation to processes and systems of oppression and domination, such as cross-disabilities) is a broad limitation and critical area for future exploration.

## Conclusions

The refined *Valuing All Voices Framework* can help inform the development of training for PE in health research, particularly in terms of building meaningful and sustainable research partnerships in areas such as critical reflexive practice, compassion, empathy, establishing trust, self-care, and addressing trauma in lived experience. If used as intended, the framework can help facilitate dialogue between researchers and patient and public partners about expectations and roles in research partnerships, while emphasizing the importance of a social justice and health equity lens in conducting transformative research. The framework can also inform development of evaluation dimensions (both formal and informal) for PE in health research. Though this study focuses on inclusion of voices traditionally less heard in health research, the refined framework should be applied in broader contexts of PE. The underlying theories and principles of trauma-informed intersectional analysis are of benefit to all teams seeking to build meaningful and trusting relationships with patient and public partners. Ongoing internal and external projects and programs examining PE in health research and healthcare services are adapting the refined *Valuing All Voices Framework* with various groups - expanding its reach and applicability and providing additional opportunities for continued refinement of the framework and its components. The *Valuing All Voices Framework* will continue to inform the advancement of inclusive and meaningful patient-oriented research by helping the broader health research community recognize both the importance of trauma-informed approaches and intersectional analysis, as well as their practical day-to-day application.

## Supplementary information

**Additional file 1: Appendix 1.** The proposed *Valuing All Voices Framework* that was discussed and revised with study participants.

**Additional file 2: Appendix 2.** Participant Interview Guide

**Additional file 3: Appendix 3.** Demographic Questionnaire

## Data Availability

The datasets generated and analyzed in the current study are not publicly available due to participant confidentiality, but are available from the corresponding author upon reasonable request.

## Abbreviations

PE: Patient engagement
OCAP™: Ownership, Control, Access, and Possession
LGBTQ2 +: Lesbian, gay, bisexual, transgender, queer (or questioning), two-spirited

## Footnotes

1 Policies, practices or procedures that result in some people receiving unequal access or being excluded, based on socially-constructed categories such as age, body size/shape, caste, class, disability, language, income, indigeneity, mental health status, name, nationality, race or ethnicity, immigration status, region, religious beliefs, sex, sex characteristics, gender identity, and sexual orientation.

2 “A set of principles that reflect First Nation commitments to use and share information in a way that brings benefit to the community while minimizing harm.” [43]

3 “The routine incorporation of new knowledge or information into environments of action that are thereby reconstituted or reorganized.” [44]
