# Supplementary figures and images for: Valuing All Voices: refining a trauma-informed, intersectional and critical reflexive framework for patient engagement in health research using a qualitative descriptive approach
Source: Res Involv Engagem. 2020 Jul 19;6:42. doi: 10.1186/s40900-020-00217-2 (PMC7370500; doi:10.1186/s40900-020-00217-2)

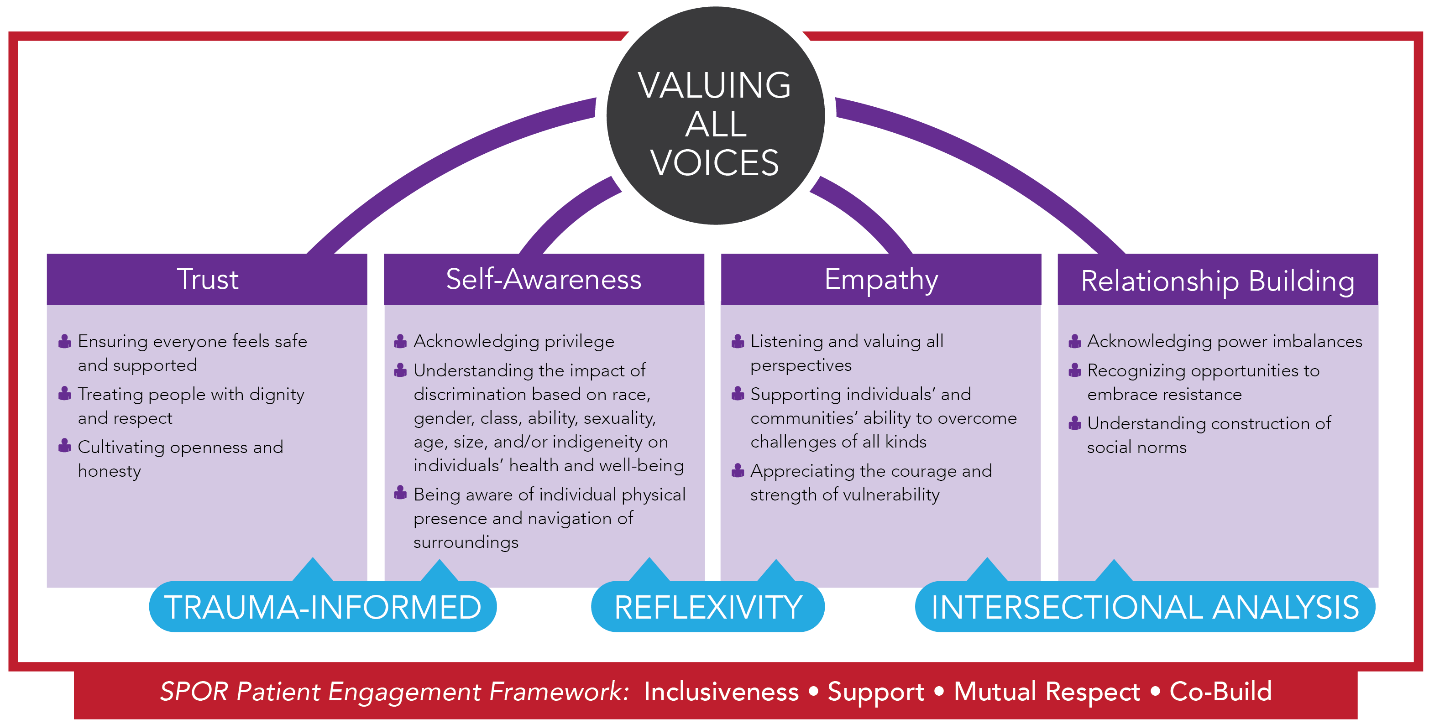

Supplement: Supplementary file 1 — Additional file 1: Appendix 1. The proposed Valuing All Voices Framework that was discussed and revised with study participants. [file 40900_2020_217_MOESM1_ESM.png]
