# Supplementary material for: Valuing All Voices: refining a trauma-informed, intersectional and critical reflexive framework for patient engagement in health research using a qualitative descriptive approach
Source: Res Involv Engagem. 2020 Jul 19;6:42. doi: 10.1186/s40900-020-00217-2 (PMC7370500; doi:10.1186/s40900-020-00217-2)
Supplement: Supplementary file 2 — Additional file 2: Appendix 2. Participant Interview Guide [file 40900_2020_217_MOESM2_ESM.docx]

**Appendix 1: Participant Interview Guide**

***Begin Recording***

Thank you for taking the time to meet to discuss the *Valuing All Voices Framework*. The purpose of this interview is to get feedback from your perspective as a person with lived experience of a health condition, or an informal caregiver for someone with a health condition.

We can begin by discussing the proposed *Framework* and how it was created (*duration and depth of interview will depend on prior discussions of the Framework and knowledge of the key informant of the principles of the Framework).*

- What patient engagement in health research is
- What the *Valuing All Voices Framework* is
- Trauma-informed practice
- Intersectionality

1. One of the important considerations for our meetings, as well as to patient and public engagement in research, is the concept of safe spaces.
   1. What does ‘safety’ mean to you?
   2. What does ‘safety’ look like to you?
   3. What does ‘safety’ feel like to you?
2. Reflexive practice questions
   1. How do my experiences of health and health systems shape my values, interests, and beliefs?
   2. How do my values, interests, and beliefs shape my experiences of health and healthcare systems?
   3. How do these experiences relate to the different ways in which I identify?
   4. How do these experiences relate to larger issues of discrimination?
   5. How do these experiences impact the way that health researchers and people with lived experience of a health issue work together on a project?
3. Components of the *Framework*
   1. Trust
      1. How do you define ‘trust’?
      2. *Introduce proposed definition:* how do you think the proposed definition compares to yours?
      3. Should anything be added or removed to the proposed definition?
      4. Is there anything else we should change about ‘trust’ in the *Framework*?
   2. Self-awareness
      1. How do you define ‘self-awareness’?
      2. *Introduce proposed definition:* how do you think the proposed definition compares to yours?
      3. Should anything be added or removed to the proposed definition?
      4. Is there anything else we should change about ‘self-awareness’ in the *Framework*?
   3. Empathy
      1. How do you define ‘empathy’?
      2. *Introduce proposed definition:* how do you think the proposed definition compares to yours?
      3. Should anything be added or removed to the proposed definition?
      4. Is there anything else we should change about ‘empathy’ in the *Framework*?
   4. Relationship building
      1. How do you define ‘relationship building’?
      2. *Introduce proposed definition:* how do you think the proposed definition compares to yours?
      3. Should anything be added or removed to the proposed definition?
      4. Is there anything else we should change about ‘relationship building’ in the *Framework*?
4. The overall *Valuing All Voices Framework*
   1. What do you like about the proposed *Framework*?
   2. What don’t you like about the proposed *Framework*?
   3. Is there anything we should add (components or otherwise) to the *Framework*?
   4. Is there anything we should remove from the *Framework*?
   5. Are there any other suggestions you have for the *Framework* that we haven’t talked about?

Is there anything else you think is important to mention?
