# Supplementary material for: Valuing All Voices: refining a trauma-informed, intersectional and critical reflexive framework for patient engagement in health research using a qualitative descriptive approach
Source: Res Involv Engagem. 2020 Jul 19;6:42. doi: 10.1186/s40900-020-00217-2 (PMC7370500; doi:10.1186/s40900-020-00217-2)
Supplement: Supplementary file 3 — Additional file 3: Appendix 3. Demographic Questionnaire [file 40900_2020_217_MOESM3_ESM.docx]

# **Appendix 3: Demographic Questionnaire**

**Descriptive Questionnaire for Participants**Please answer the following. All responses will be kept confidential.

1. What is your age? _______________
2. What is your gender?
   - Man
   - Woman
   - Transgendered
   - Two-spirited
   - Other: ____________________
   - Prefer not to answer
3. With which of the following do you have experience? (Choose all that apply)
   - A personal health condition requiring some form of support
   - Caregiving for another person with a health condition
   - Accessing healthcare for a health condition (emergency room, hospital, walk-in, doctor, or other healthcare professional)
   - Being unable to access healthcare for a health condition
   - Participating in health research as a participant or patient (not including this study)
4. With which groups/communities do you identify? (Choose all that apply)
   - Person with a disability
   - LGBTQ2+
   - Indigenous
   - Immigrant
   - Refugee
   - Hearing impaired or deaf
   - Visually impaired or blind
   - Other: _________________________________________________
